# Supplementary material for: Multiplexed, Sequential Secretion Analysis of the Same Single Cells Reveals Distinct Effector Response Dynamics Dependent on the Initial Basal State
Source: Adv Sci (Weinh). 2019 Mar 13;6(9):1801361. doi: 10.1002/advs.201801361 (PMC6498135; doi:10.1002/advs.201801361)
Supplement: Supplementary file 1 — Supplementary [file ADVS-6-1801361-s001.pdf]

Copyright WILEY-VCH Verlag GmbH & Co. KGaA, 69469 Weinheim, Germany, 2019.

ADVANCED SCIENCE Open Access

Supporting Information

for *Adv. Sci.,* DOI: 10.1002/advs.201801361

Multiplexed, Sequential Secretion Analysis of the Same Single Cells Reveals Distinct Effector Response Dynamics Dependent on the Initial Basal State

Zhuo Chen, Yao Lu, Kerou Zhang, Yang Xiao, Jun Lu, and Rong Fan**

Supporting Information

Multiplexed, sequential secretion analysis of the same single cells reveals distinct effector response dynamics dependent on the initial basal state

Zhuo Chen[1#], Yao Lu[1,2#*], Kerou Zhang[1], Yang Xiao[1], Jun Lu[3,4], and Rong Fan[1,3,4,5]*

[1] Department of Biomedical Engineering, Yale University, New Haven, CT 06520, USA

[2] Department of Biotechnology, Dalian Institute of Chemical Physics, Chinese Academy of Sciences, Dalian, Liaoning, 116023 China

[3] Department of Genetics, Yale School of Medicine, New Haven, CT 06520, USA
[4] Yale Stem Cell Center, New Haven, CT 06520, USA

[5] Yale Cancer Center, New Haven, CT 06520, USA

# these authors contribute equally to this work.

* corresponding authors, Y.L.(luyao@dicp.ac.cn) and R.F. (rong.fan@yale.edu)

Yao Lu, Ph.D.
Department of Biotechnology
Dalian Institute of Chemical Physics, Chinese Academy of Sciences
457 Zhongshan Road, Dalian, Liaoning, 116023 China
Email: luyao@dicp.ac.cn

Rong Fan, Ph.D.
Department of Biomedical Engineering
55 Prospect St, MEC 213
New Haven, CT 06520
U.S.A.
Email: rong.fan@yale.edu

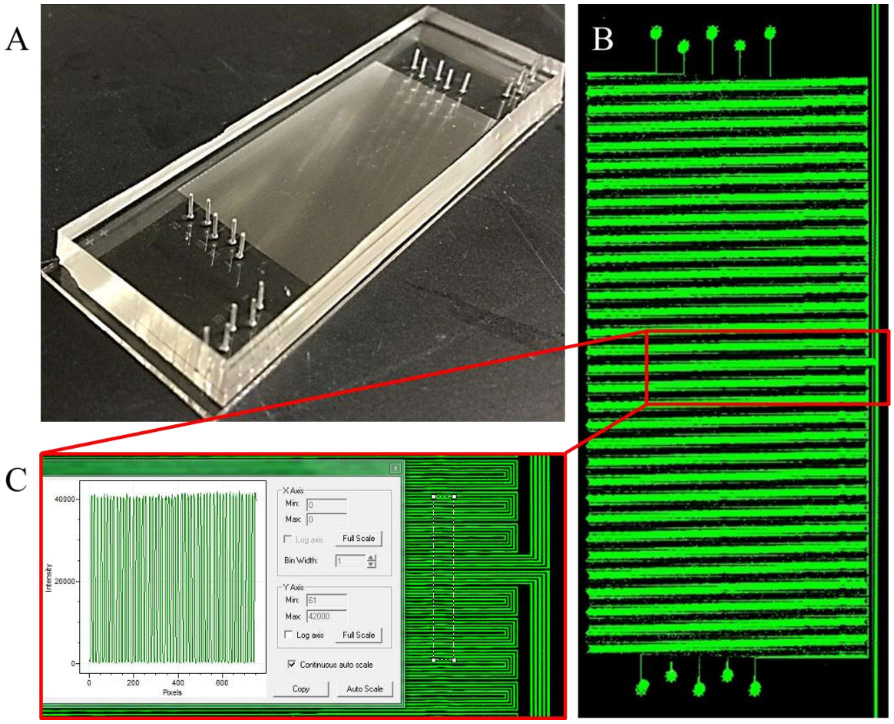

Figure S1. Evaluation of the protein coating uniformity flow-patterned with antibody barcode microchannel. A) Optical image showing the PDMS microchip for antibody flow patterning; B) whole glass slide fluorescence scanning; (C) magnified view of fluorescence intensity across the flow patterned poly-L-lysine slide revealed excellent uniformity of the immobilized proteins (FITC-BSA) in two separated paths, which ensures the validity of using this high-density barcode array technology to assess single cell heterogeneity.

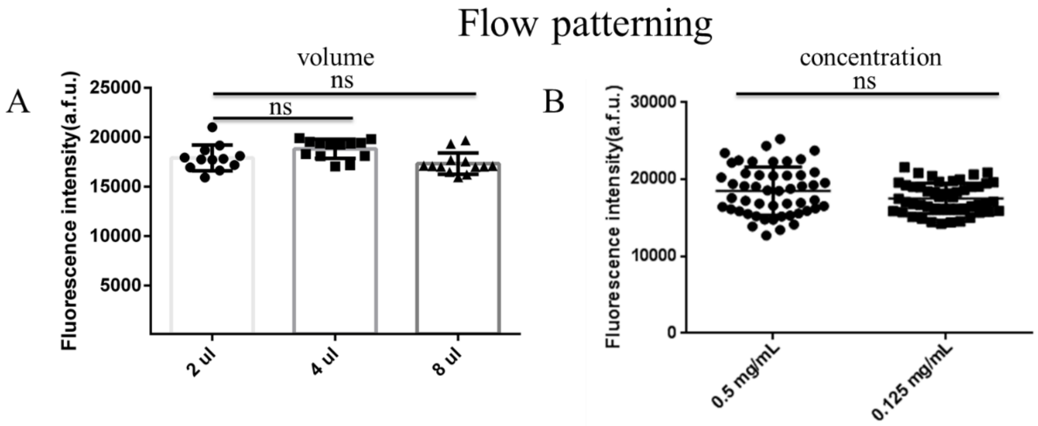

Figure S2. Evaluation of the antibody flow patterning conditions. A, different volume of antibodies; B, different antibody dilutions. The results showed no significant differences with varied flow patterning conditions.

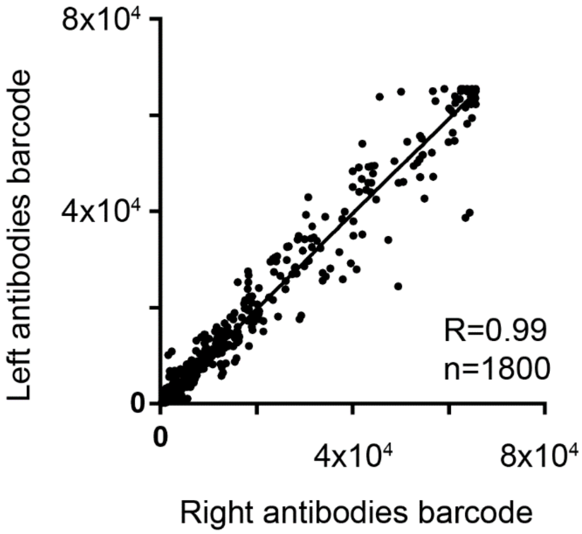

Figure S3. Characterization of the fluorescence detection results based on left and right antibodies barcode, which showed nice correlation between them.

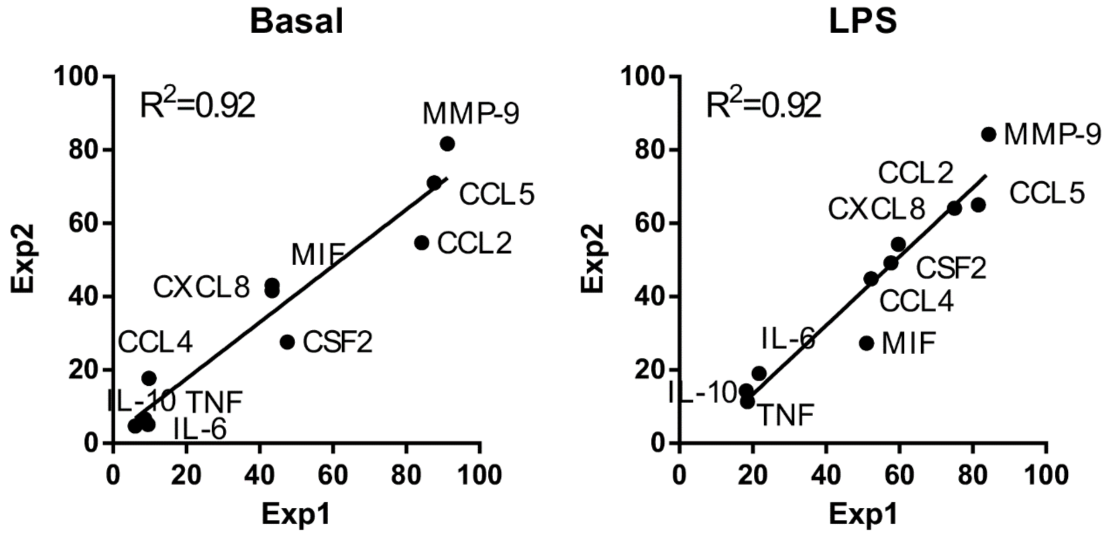

Figure S4. Validation of reproducibility of the single-cell barcode chip. Cytokine secretion frequencies (10 proteins) with U937 derived macrophages from two independent experiments (basal and LPS) showed high, linear correlation (R^2 = 0.92).

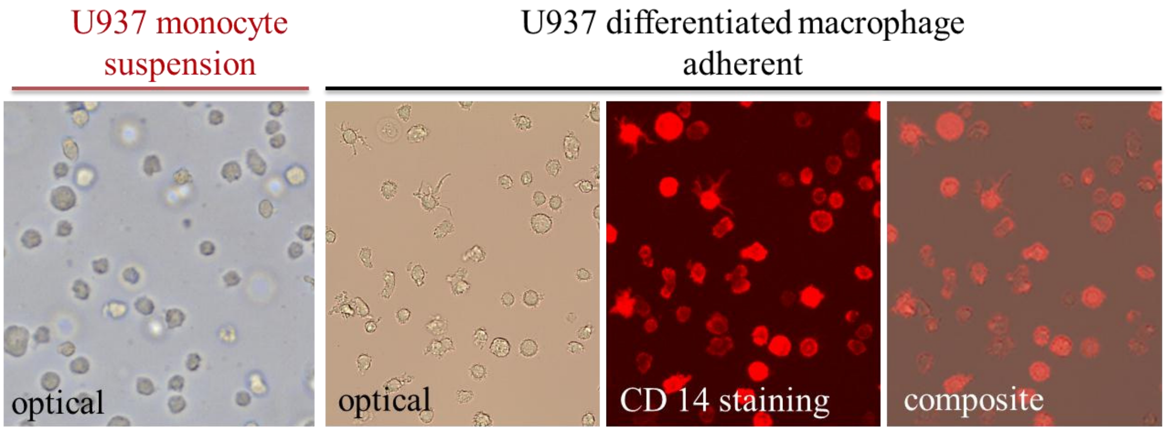

Figure S5. Images showing differentiation of U937 monocyte cells. Monocyte cells became adherent when differentiated into macrophage cells with 50 ng/mL PMA for 48 hours.

A

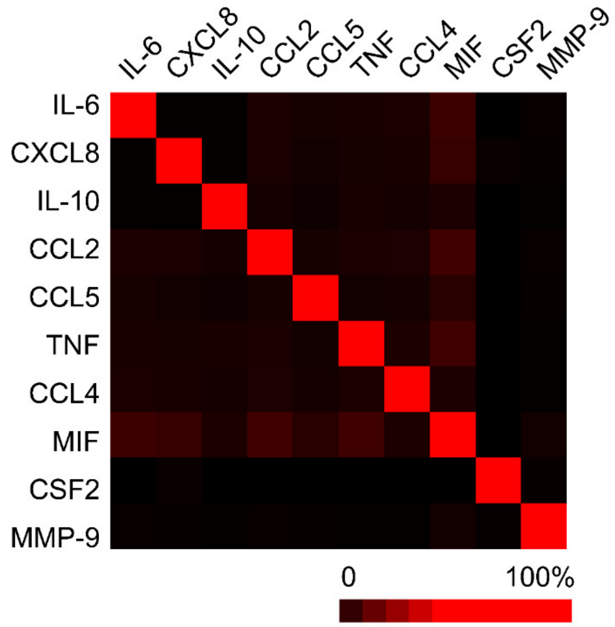

B

Proteins	IL-6	CXCL8	IL-10	CCL2	CCL5	TNF	CCL4	MIF	CSF2	MMP-9
IL-6	100.0%									
CXCL8	1.1%	100.0%								
IL-10	0.9%	0.9%	100.0%							
CCL2	5.6%	5.4%	4.0%	100.0%						
CCL5	4.4%	3.6%	2.9%	4.4%	100.0%					
TNF	4.7%	4.5%	4.9%	5.2%	3.7%	100.0%				
CCL4	5.2%	4.7%	4.0%	5.8%	4.2%	5.7%	100.0%			
MIF	11.8%	10.7%	5.6%	12.9%	7.7%	12.4%	5.6%	100.0%		
CSF2	0.3%	1.9%	0.2%	0.2%	0.2%	0.2%	0.1%	0.3%	100.0%	
MMP-9	1.7%	1.2%	0.7%	1.6%	1.3%	0.7%	0.6%	3.7%	1.2%	100.0%

Figure S6. Cross-reactivity check for all proteins (A: heatmap; B: raw values). Most of the antibodies used in this study are monoclonal antibodies to ensure good specificity and

reduce cross reactivity. The test was conducted by spiking a single antigen (recombinant protein) in a solution applied to a full capture antibody microarray containing all capture antibodies, followed by detection with a mixture of all detection Ab

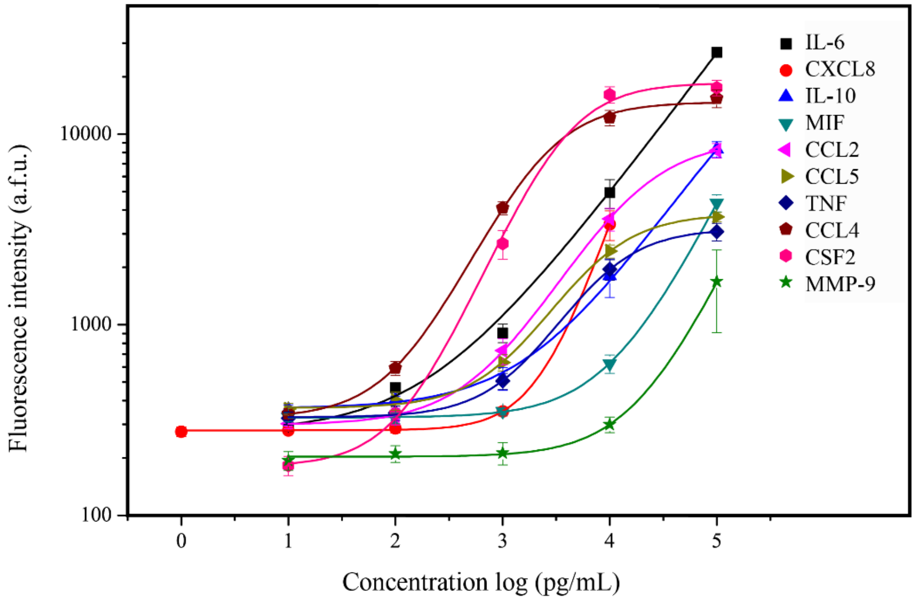

Figure S7. Calibration curves obtained with corresponding recombinant proteins (x, y axes: log-scaled, the intensities were averaged from 16 spots for each data).

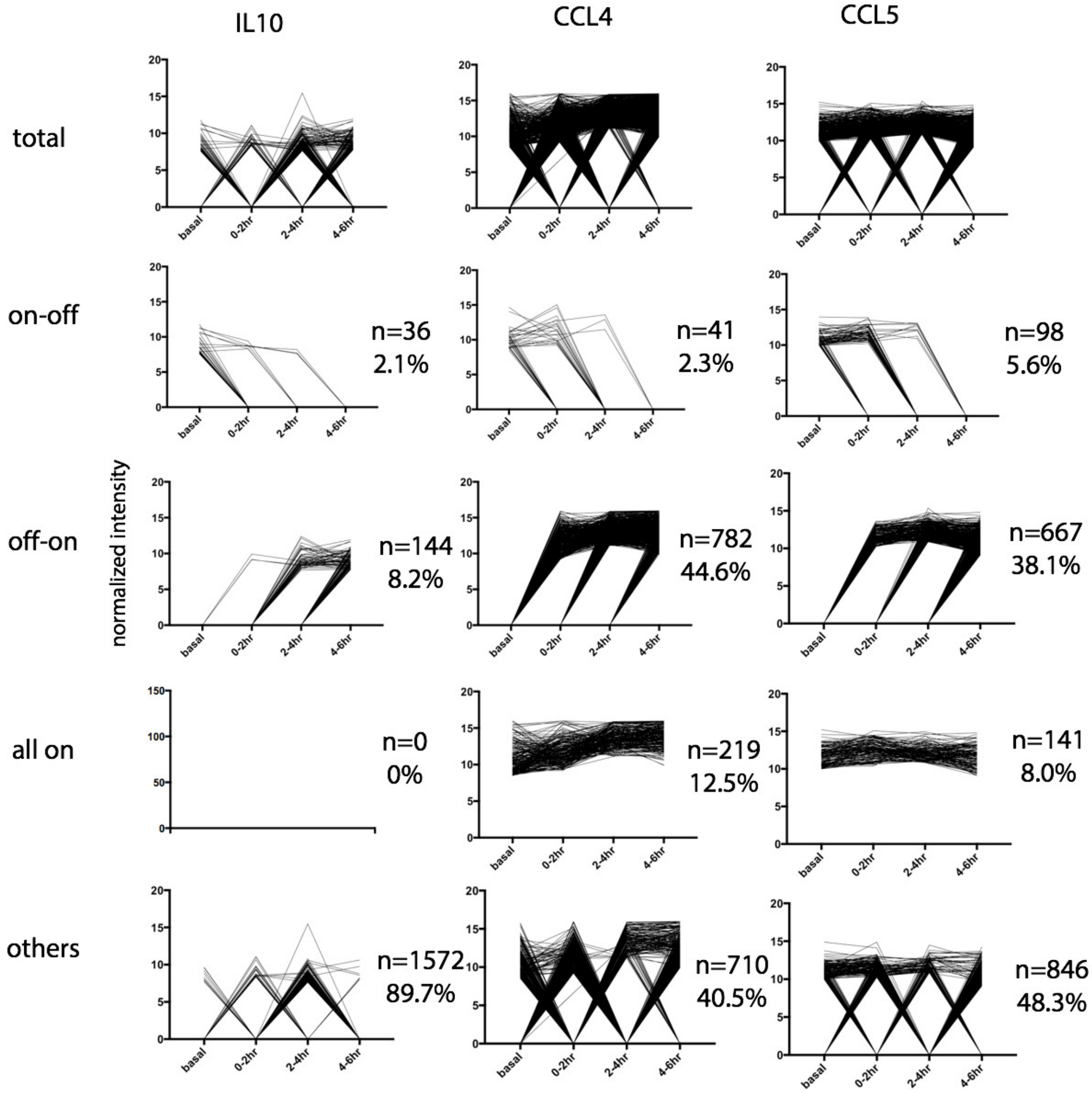

Figure S8. Classification of single cells based on their protein secretion dynamics.

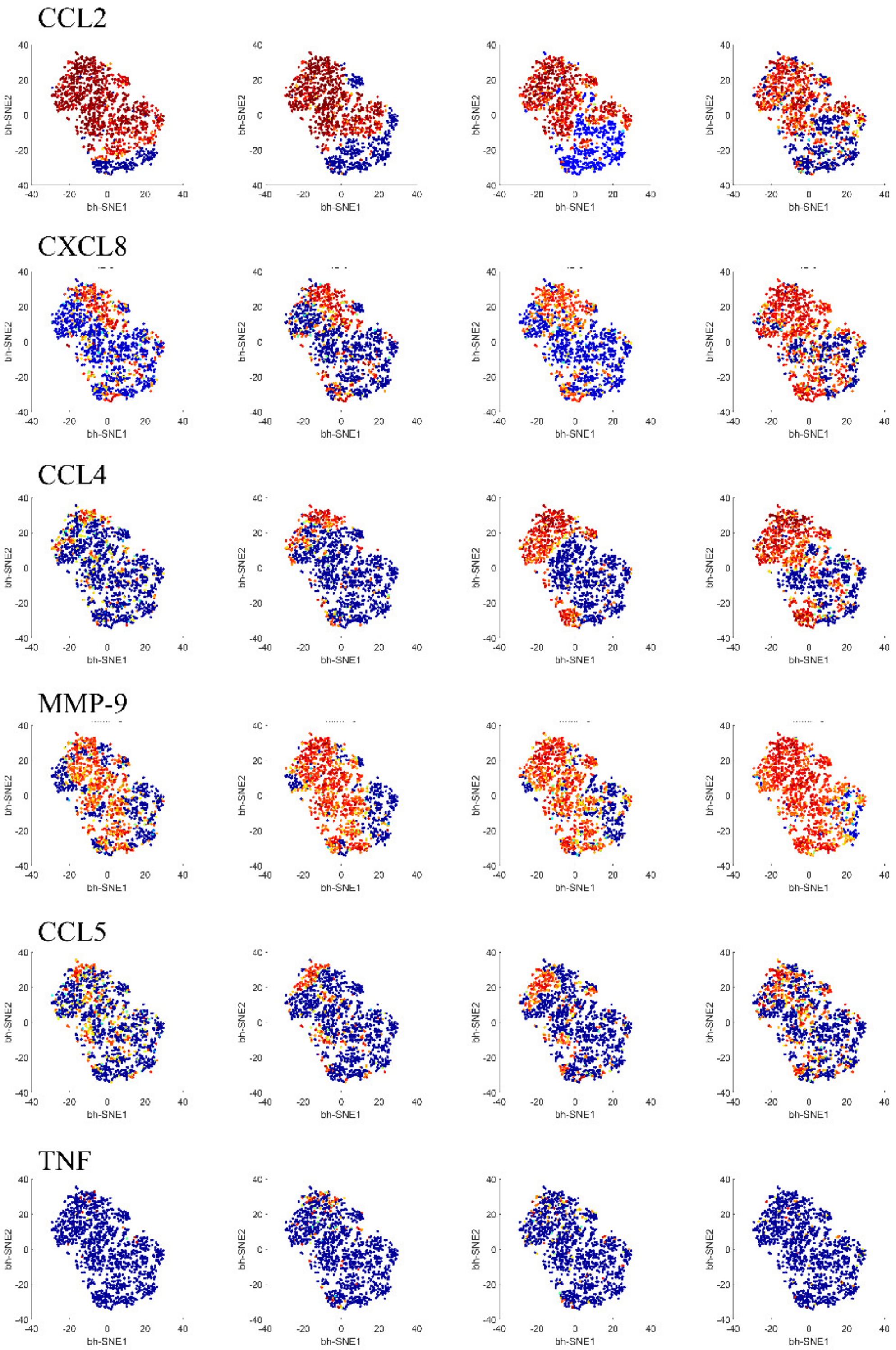
CCL2
CXCL8
CCL4
MMP-9
CCL5
TNF
bh-SNE1
bh-SNE2

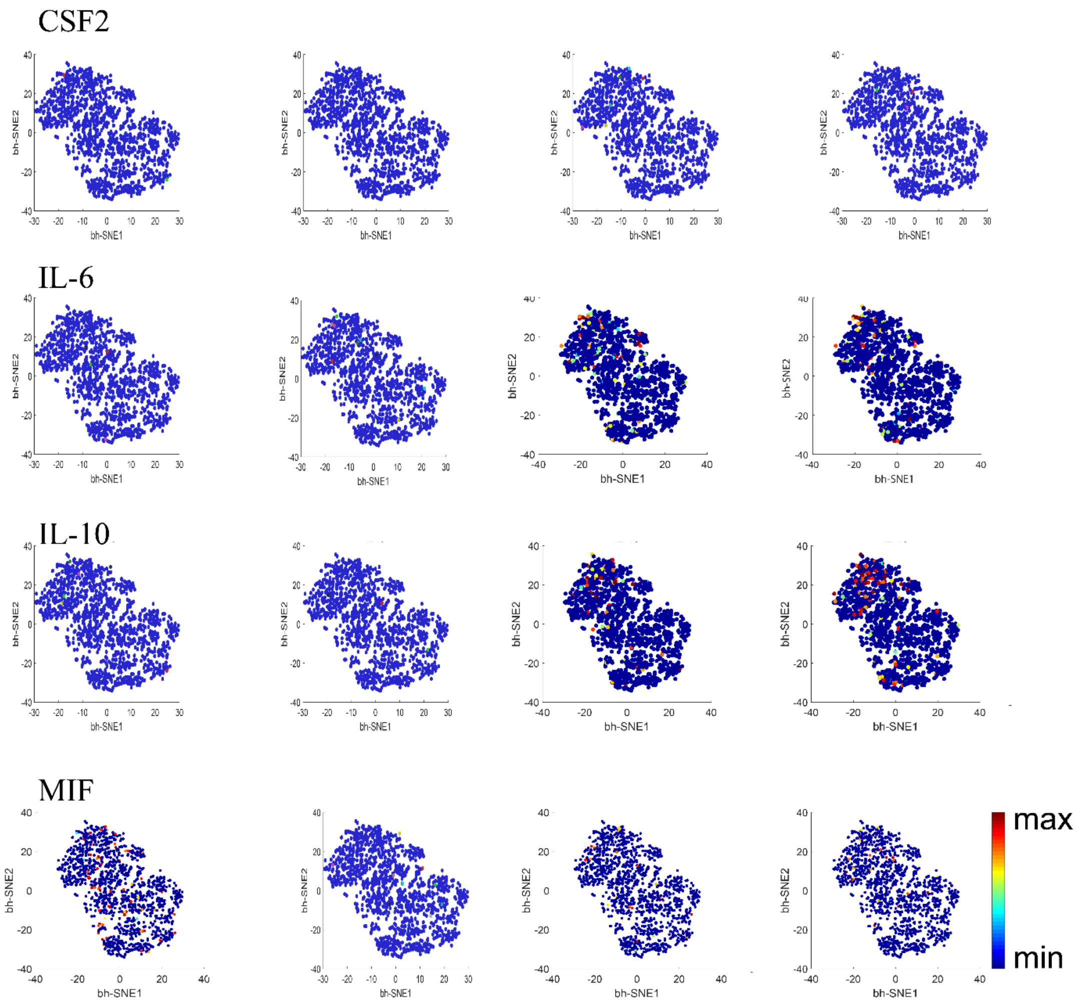

Fig. S9 viSNE maps showing the distribution of each protein at different time points.

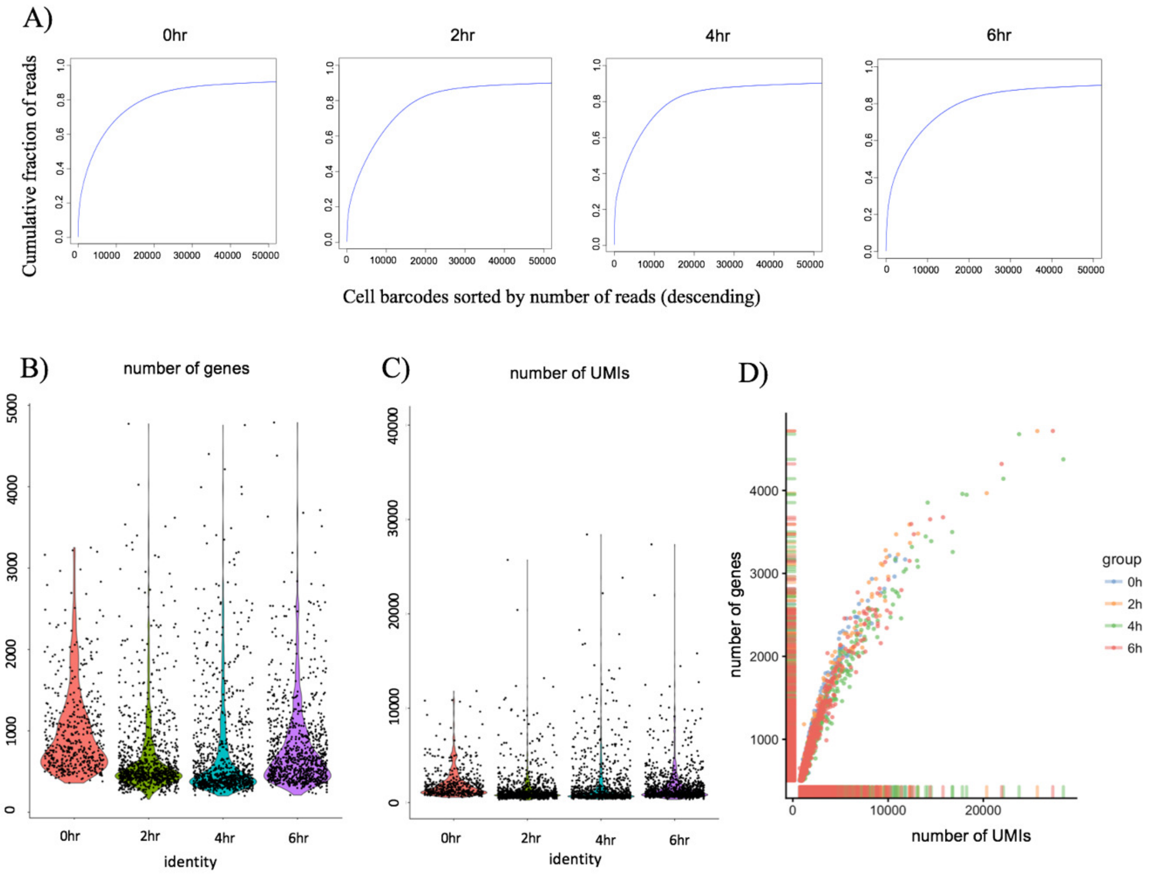

Figure S10. Quality control of single-cell RNA-Seq data. A) Saturation curve of sequencing reads. The deflection point of the curve shows the approximate number of cells sequenced. B) Violin plots showing distribution of number of genes detected from each single cell. C) Violin plots showing distribution of number of UMIs detected from each single cell. D) correlation between number of genes and UMIs detected from each single cell.

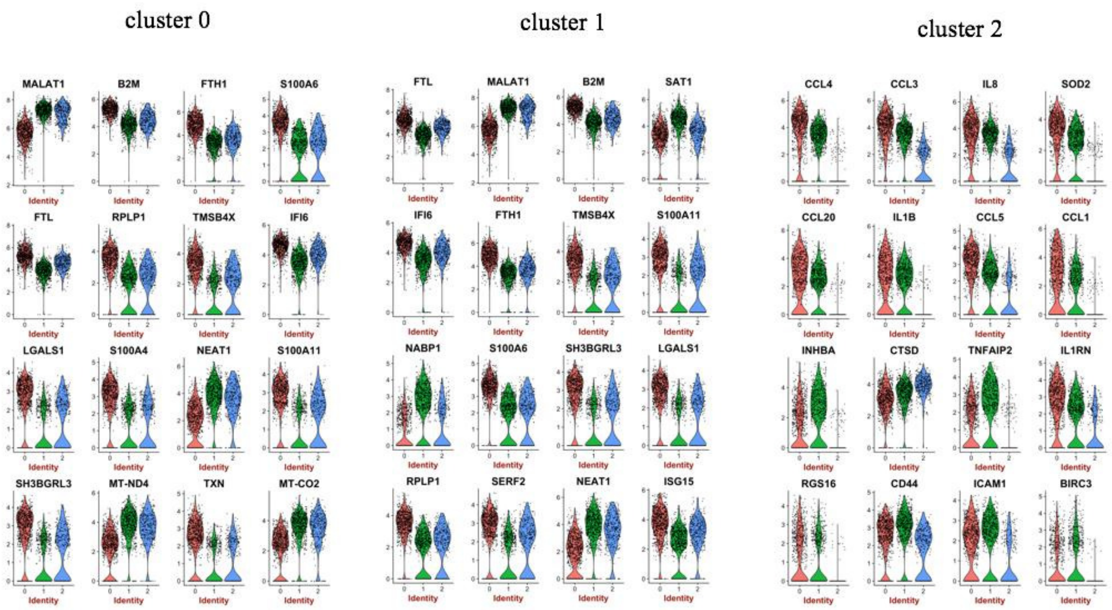

Figure S11. Top 16 marker genes of the three clusters, respectively.

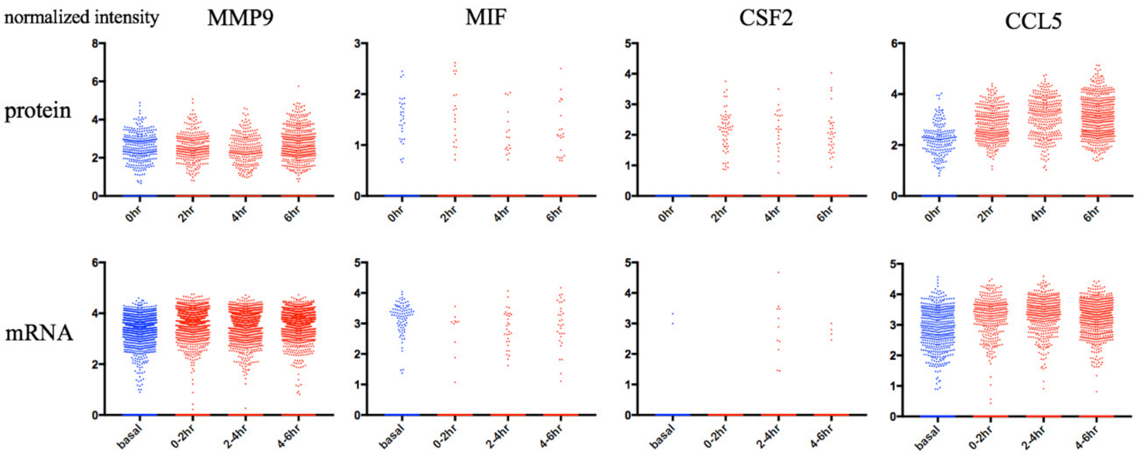

Figure S12. Comparative study of single-cell RNA expression and protein secretions.

Supplementary Table S1: Summary of antibodies (name, clone, company, catalog) used in this study (most are monoclonal antibodies).

Protein	**Capture antibody Isotype/clone/vendor/catalog**	**Detection antibody Isotype/clone/vendor /catalog**
IL6	Rat IgG2a, κ /MQ2-39C3/Biolegend/501204	Rat IgG1, κ /MQ2-13A5/Biolegend/ 501102
CXCL8	Mouse IgG1, κ /H8A5/Biolegend/511502	Mouse IgG1, κ /E8N1/Biolegend/ 511402
IL10	Rat IgG2a, κ/JES3-12G8/Biolegend/ 501504	Rat IgG1, κ /JES3-9D7/Biolegend/ 501402
MIF	Mouse IgG1 Kappa/2A10-4D3/Abnova/H00004282-M01	Mouse IgG1 Kappa/2A10-4D3/Abnova/H00004282-M01
CCL2	Mouse IgG1, κ /5D3-F7/Biolegend/502607	Armenian Hamster IgG /2H5/Biolegend/ 505902
CCL5	Mouse IgG2b kappa/VL1/Invitrogen/AHC1052	Mouse IgG1 /21418/RD/MAB678
TNF	Mouse IgG1, κ /Mab11/Biolegend /502902	Mouse IgG1, κ /MAb1/Biolegend/502802
CCL4	Mouse IgG1 kappa/A174E 18A7/Invitrogen/AHC6114	Mouse IgG2B /24006/RD/MAB271
CSF2	Rat IgG2a, κ /BVD2-23B6/Biolegend/502202	Rat IgG2a, κ /BVD2-21C11/Biolegend/502304
MMP9	Mouse IgG1 /36020/RD/MAB936	Goat IgG /polyclonal/RD/BAF911

Supplementary Table S2: Reagents used in single-cell RNA-Seq experiment.

Reagents	Supplier	Catalog #
Barcoded microparticles	Chemgenes	N/A
Maxima H-Reverse Transcriptase	Thermo	EP0753
dNTP mix	Clontech	639125
RNase inhibitor	Lucigen	30281-2
Perfluorooctanol	Sigma	370533
Exonuclease I	NEB	M0293L
KAPA Hifi HotStart Readymix	KAPA BioSystems	KK2602
Nextera XT DNA sample preparation kit	Illumina	FC-131-1096
Ampure XP beads	Backman Coulter	A63882
